# Indigenous patient experiences of returning to country: a qualitative evaluation on the Country Health SA Dialysis bus

**DOI:** 10.1186/s12913-018-3849-4

**Published:** 2018-12-29

**Authors:** Jessica Conway, Sharon Lawn, Susan Crail, Stephen McDonald

**Affiliations:** 1 Sydney Local Health District, Sydney, NSW Australia; 2 Flinders Human Behaviour and Health Research Unit, Department of Psychiatry, Flinders University, Adelaide, Australia; 3 Central Adelaide Renal and Transplantation Service, Adelaide, Australia; 4 Country Health SA Local Health Network, SA Health and Medical Research Institute, Adelaide Medical School, University of Adelaide, Adelaide, Australia

**Keywords:** Chronic kidney disease, Indigenous health, Haemodialysis, Remote dialysis access, Mobile Dialysis

## Abstract

**Background:**

Rates of End-Stage Kidney Disease among Aboriginal and Torres Strait Islander (Indigenous) Australians in remote areas are disproportionately high; however, haemodialysis is not currently offered in most remote areas. People must therefore leave their ‘Country’ (with its traditions and supports) and relocate to metropolitan or regional centres, disrupting their kinship and the cultural ties that are important for their wellbeing. The South Australian Mobile Dialysis Truck is a service which visits remote communities for one to two week periods; allowing patients to have dialysis on ‘Country’, reuniting them with their friends and family, and providing a chance to take part in cultural activities. The aims of the study were to qualitatively evaluate the South Australian Mobile Dialysis Truck program, its impact on the health and wellbeing of Indigenous dialysis patients, and the facilitators and barriers to using the service.

**Methods:**

Face to face semi-structured interviews were conducted with 15 Indigenous dialysis patients and 10 nurses who had attended trips across nine dialysis units. Realist evaluation methodology and thematic analysis established patient and nursing experiences with the Mobile Dialysis Truck.

**Results:**

The consequences of leaving Country included grief and loss. Barriers to trip attendance included lower trip frequencies, ineffective trip advertisement, lack of appropriate or unavailable accommodation for staff and patients and poor patient health. Benefits of the service included the ability to fulfil cultural commitments, minimisation of medical retrievals from patients missing dialysis to return to remote areas, improved trust and relationships between patients and staff, and improved patient quality of life. The bus also provided a valuable cultural learning opportunity for staff. Facilitators to successful trips included support staff, clinical back-up and a co-ordinator role.

**Conclusions:**

The Mobile Dialysis Truck was found to improve the social and emotional wellbeing of Indigenous patients who have had to relocate for dialysis, and build positive relationships and trust between metropolitan nurses and remote patients. The trust fostered improved engagement with associated health services. It also provided valuable cultural learning opportunities for nursing staff. This format of health service may improve cultural competencies with nursing staff who provide regular care for Indigenous patients.

**Electronic supplementary material:**

The online version of this article (10.1186/s12913-018-3849-4) contains supplementary material, which is available to authorized users.

## Background

While Australia is a vast continent, the majority of the population dwell close to its coastlines in urban and regional centres. By contrast, many Aboriginal and Torres Strait Islander (Indigenous) Australians live in remote areas where they strive to maintain their communities and cultural traditions. As a consequence, health services and other infrastructure can be limited. In such remote locations, the unique and rugged landscape and relative isolation pose significant access challenges. This competes with effective provision of care for Indigenous Australians with chronic conditions. Compounding this, the rates of end-stage kidney disease (ESKD) among Indigenous Australians are disproportionately higher than non-Indigenous Australians, in particular in remote areas, where the incidence is up to 18 to 20 times that of metropolitan areas [1]. The determinants underlying this are complex, ranging from low birthweight through high rates of infectious illnesses to very high rates of diabetes and other non-communicable diseases [2–4].

The most common form of kidney replacement therapy is haemodialysis [5–7], which is required three times a week. Most Indigenous Australians with ESKD attend in-centre haemodialysis in large urban settings as provision of dialysis in most remote communities is not currently provided, reflecting distance, sparse population and associated costs with providing infrastructure and staffing [8]. For this reason, 78% of indigenous patients relocate to metropolitan centres for dialysis [9]. For some communities such as those in the Anangu Pitjantjatjara Yankunytjatjara (APY) lands in the far North West corner of South Australia (SA), the distance is as far as 1835 km by road to Adelaide. The communities the truck visits are located in a vast area of arid desert in the far northwest of SA [10]. Most communities visited are “owned” by the “traditional owners” but Marla and Coober Pedy are small regional centres. Roads are typically gravel, with mobile phone coverage limited to townships. As such, dialysis access for many from these areas has been restricted to centres many hours drive away, further magnifying the dislocation from ‘Country’ [11]. These communities each have between two and eight patients who have relocated for haemodialysis.

This dislocation from “Country” and family is associated with a sense of loss and disempowerment [12], to the detriment of social and emotional wellbeing [9]. For Indigenous Australians, “Country” is more than a reference to land or a home; it is a unique connection to land, similar to a family bond. “Country” has associations with spirituality, tradition and ownership as the foundation of social structures, systems and cultural practices are entwined with the land. This relationship is vital for identity, health and wellbeing [13]. The absence of treatment options in communities on Country discourages Indigenous people in remote areas from seeking treatment [9, 12]. This may be associated with non-attendance to dialysis sessions [14], leading to adverse health outcomes [15].

**Fig. 1:**
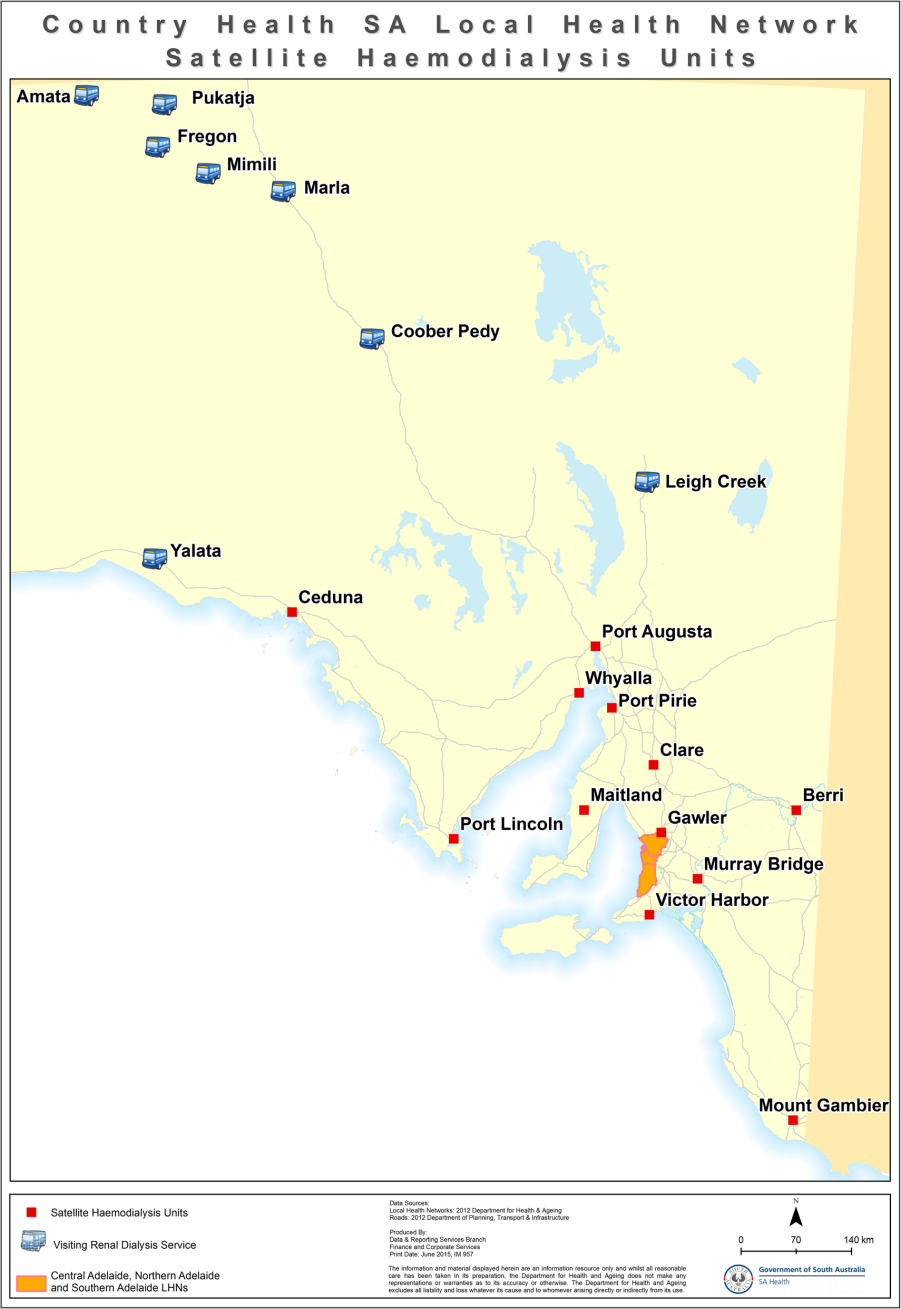
Towns serviced by the dialysis bus and relative distance to Adelaide (Capital of SA). Source: SA Health, Data and Reporting Services Branch, Finance and Corporate Services. Country Health SA Local Health Network Satellite Haemodialysis Units. Adelaide: Author; 2015

Reuniting patients with their communities improves Indigenous Australians’ health and wellbeing [16]. The holistic view of health of Indigenous peoples from many nations across the globe is well documented [17–19]. This includes the importance of the connection to land, the interconnection of cultural and environmental aspects of the health of the community and the wellbeing of the individual [20]. As part of a National reform in health care, recent models have tried to address this importance, bringing access to health care to the communities by way of mobile health facilities [21]. Across the globe there are mobile dialysis units that work either to provide respite and holidays of dialysis patients [22] or provide access to haemodialysis for rural and remote patients [23]. There are three dialysis busses in Australia that assist Indigenous patients in visiting their homelands, the Purple Truck [24] and the Country Health SA dialysis bus [25]. Another Northern Territory dialysis bus is not currently in use [26]. In Canada, a mobile service provides haemodialysis access to two towns in rural western Alberta by driving between the towns so that patients there do not need to relocate [23]. In order to provide a connection for the dislocated Indigenous patients on dialysis, the Country Health SA mobile dialysis truck (hereinafter referred to as ‘the dialysis bus’) has been visiting remote SA communities since 2014 (see Fig. 1). It has three dialysis chairs and a wheelchair lifter to assist access for all patients [27]. This allows Indigenous dialysis patients forced to relocate for dialysis to visit their home communities for significant events (such as funerals and cultural ceremonies) and to spend time with family and friends [5]. The bus is managed by a co-ordinator and team at Country Health SA (a state Government funded health service) with assistance from Nurse Unit Managers from dialysis centres across the state. Staff assist patients in arranging their own transport to their communities. Central funding covers staff and bus running costs but not patient travel and accommodation expenses. The dialysis bus is driven together with a support vehicle(s) for staff and patient transport to the relevant community. The support vehicle(s) are also used to collect patients from their homes in the community during the visit. The dialysis bus has 3 chairs, and the capacity to support dialysis for up to 12 patients on any one trip. Trips generally run for two weeks but one to four week trips have also occurred. Staff are accommodated at nearby nurse quarters or hotels depending on the community’s capacity. The communities vary in their distance from Adelaide, from 990 to 1885 km by road. Two to four nurses attend each bus trip at one time, and generally work six out of seven days. This study is the first to evaluate this type of service.

## Methods

The target population were Indigenous haemodialysis patients and staff who had attended trips on the dialysis bus. The researcher made contact with each Nurse Unit Manager providing information about the study who then approached patients who had participated in previous bus visits with information to gauge interest. Following this, the researcher approached interested patients to consent for interview. Recruitment stopped once data saturation was achieved with replication of codes.

Nursing staff were recruited via email invitation. Potential participants were identified by key staff informants who included the manager of the dialysis bus, prior bus co-ordinator, and nurse unit managers. Participants included staff who had experienced multiple trips, those who had decided not to return on subsequent trips or who encountered new challenges on trips that required troubleshooting, thereby exploring a breadth of experiences. After responding to an invitation an interview was conducted at their convenience. There were 15 patients and 10 staff participants from a variety of metropolitan and regional renal dialysis units. Of the patients, five were male and 10 were female; and, of the staff, two were male and eight were female.

In-depth interviews were used to investigate the perspectives of staff and patients on the dialysis bus. Interviews with patients were conducted in a story-telling or ‘yarning’ manner. Yarning is a method of information sharing in a relaxed, informal setting where the subject recounts experiences in a story-telling manner [28]. Yarning provides an avenue for performing a semi-structured or unstructured interviews which provide a ‘holistic understanding’ of the participants’ experiences [29]. It has been regarded as a culturally appropriate mode of data gathering from Indigenous people [16]. Interview questions to guide the yarn were subject to ongoing review throughout data collection. These questions are provided as a Additional file 1. All interviews occurred face-to-face. All staff consented to voice recording. After the researcher noted a decline in the number of patients consenting to their interview being voice recorded, it was felt that patient voice recording would negatively affect rapport. All further patient interviews were conducted with the researcher taking notes by hand (using short-hand to capture quotes) during interviews. Member checking was not possible directly after transcription, given the long distances travelled to reach regional patients in Port Augusta and Alice Springs.

Exploratory research methodology underpins this study as it sought to discover and generate theories on how the bus benefits patients and staff [30], developing theory from data, undergoing continuous data analysis throughout the process of collection [30]. New concepts and generalisations were brought from the data following open coding [31], and then organised into axial and selective codes. Exploratory methodology, in particular, was most appropriate for this study, given that there has been no prior research evaluating this type of Indigenous health service.

Using exploratory methodology, this study sought to describe how different factors may impact on service delivery, such as individual capacities, interpersonal relationships between the patient and the nurses and communities, and the infrastructure and systems that support or undermine the workings of the dialysis bus. Given this is an area of research which has not been previously studied, the chosen methodology allowed the examination of the barriers and facilitators that patients and staff experience in using the service, and given the social context of the program, the methodology also acknowledged that the interpretations of the program’s participants, namely the staff and patients, were integral to exploring its benefits.

Data analysis for this study was performed using thematic analysis [32]. Following transcription of interviews by the lead researcher, two researchers (JC and SL) coded six interviews separately (three patient and three staff), to improve validity of commonly identified codes and ensure codes were not missed. Axial coding was completed with mind mapping software LucidChart Inc. [33], grouping inter-related themes. Final stages of analysis connected themes with direct quotes from the data against notes taken on emphasised words and vocal tone providing data for triangulation [32].

## Results

**Fig. 2:**
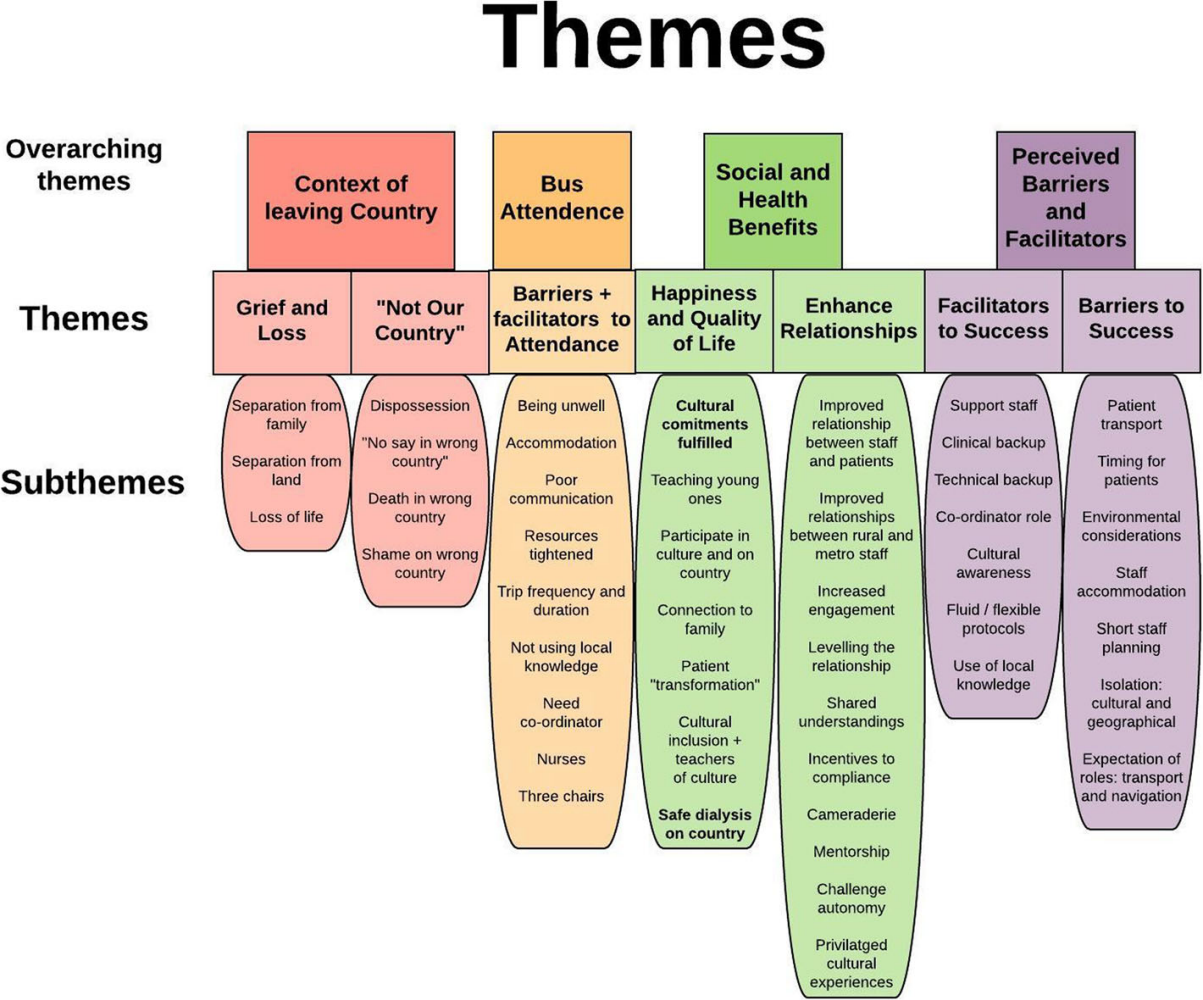
Themes arising from the data

The results are presented within four overarching themes, further divided into seven smaller themes, as shown in Fig. 2. The four overarching themes include ‘Context of Leaving Country’, ‘Bus Attendance’, ‘Social and Health Benefits’, and ‘Perceived Barriers and Facilitators’. Within ‘Context of Leaving Country’, the themes are ‘Grief and Loss’ and ‘Not Our Country’, the last being a powerful theme raising several issues including dying in the wrong Country and the shame associated with dialysing in the wrong Country. Within ‘Bus Attendance’ the themes are ‘Barriers to Attendance’ and ‘Facilitators to Attendance’. As more barriers were identified, these two themes will be presented together. Within ‘Social and Health Benefits’, the themes are ‘Happiness and Quality of Life’, a dominant theme reflecting how cultural commitments are fulfilled with participation on country and connection to family, and ‘Enhanced Relationships’. ‘Enhanced Relationships’ is an important theme as it includes the findings of improved trust and shared understandings between patients and staff who spent time on bus trips. The last overarching theme of ‘Perceived Barriers and facilitators’ includes the themes ‘Facilitators to Success’ and ‘Barriers to Success’.

### Theme 1: Grief and loss

The devastating consequences of having to relocate away from Country were evident in interviews with patients. Separation from the land and its people was particularly prominent. Grief from missing family was expressed, and returning to Country meant they could be reunited. Patients described activities on land they missed which had cultural significance. The loss of life associated with chronic kidney disease in family members saddened patients who experience first-hand the inequitable affliction of kidney disease. Despite the risks of missing haemodialysis, one patient explained how he often misses treatments to travel to his Country to take part in local meetings.

#### Grief and loss: Quotes

P2: “*Some of us have grandchildren and we can’t teach them in Port Augusta and Adelaide coz we’re not in the right Country.”*

P3: “*I’ll be happy to see my family because I miss them. That’s what we always want … I had to move here. I used to get homesick.”*

P2: “*I miss out on hunting Kangaroo, showing kids the Country, teaching our children about our dreaming.”*

P4 *“I had 2 nieces on dialysis here. I knew the troubles. They both passed away. I’m older than both of them. They too young”*.

### Theme 2: “Not Our Country”

This theme reflects the implications for dialysing on the “wrong” Country for patients and their communities. Moving to a new Country not only created an inability to contribute on issues regarding land and culture, but also the displacement of their voices in their communities by other Indigenous groups. Patients also felt they had displaced others from a dialysis position in their home town by occupying a dialysis chair that might otherwise been used by a local member starting dialysis. The significance of dying on Country was also raised as an issue for moving away from Country.

#### “Not Our Country”: Quotes

*P1: “Here I am powerless, I have no say. It’s not my Country. I need to get back to my own Country. There are other tribes running my Country. I feel like we’re not part of the community here. I can’t go to there”.*

*P2: ‘Here, we have taken someone else’s chair. We feel shame because it is not our Country here. What is someone else is from here and they can’t come back from Adelaide coz there’s not chairs here. I’ve taken up that chair. [We] feel a lot of shame for that.’*

*P1: ‘The elder’s like to go back home to die, our spirit. My spirit is happier in our Country. We End up in hospital and we want to run away. Our elder want to run away coz he’s very spiritual. In spirit world, if you go to the wrong Country you’re not welcome, and you gotta leave’.*

### Theme 3: Barriers and facilitators to attendance

‘Barriers and Facilitators to Attendance’ identified issues which precluded or assisted patients attending bus trips. These included being unwell; not having appropriate accommodation; poor communication meaning some patients were unaware of trips altogether and a sense that resources were being tightened. Patients identified that being unwell meant it was too unsafe to attend trips and wanted to be well enough to participate in trips. Nursing staff had experienced seeing patients attend dialysis more frequently and appear to better look after themselves in order to be well enough for the time set for a bus trip.

#### Bus attendance: Quotes

P9: “*I was too sick before, I missed out. I told ‘em I can’t go.”*

S2: *“if you’re stable on dialysis you could go on to the communities so it made a lot of people come to dialysis and they knew that this as an incentive”.*

Patients and staff identified how some patients were missing out on trips as they were unable to organise transport or accommodation. Most patients were able to stay with their families, however some either had no family left in their communities or their families were anxious to have them stay due to their illness, worried that they will become unwell on Country. Many staff identified difficulty obtaining transport under patients’ financial constraints further compounded by time limitations around trips. The dialysis bus staff worked to access funds from other areas to facilitate transport for patients, but most patients relied on family for transport.

#### Bus attendance: Quotes

*P1: ‘I got two aunties in Adelaide and they been renal for 20 years. They like to come back to visit Coober Pedy but they got no accommodation. So they can’t go, they gonna stay in a hotel?… They are trying to raise that issue but nobody listens to us’*.

*S1: ‘[One patient] wants to go up there and get a Nunkarri (Indigenous Healer), he wants to catch emus, he wants to just catch up with people and revisit, and none of the family are willing to take him coz they say ‘you’re too sick’ ‘we’re not gonna take you that far, even though the bus is up there they’re not comfortable doing that’.*

S7: *“They often need to have the finances to buy the bus ticket, if it’s not funded by the community that they’re from which can be a limitation”*.

S6 *“A time they lost [a patient], they relied on family for transport.. there were rumours that she was in Fregon. … [She] Ended up missing two lots of dialysis”.*

The sense that the resources were being tightened as reflected by fewer trips in the most recent year was demoralising for patients who wanted more opportunities to visit their communities. Most patients expressed the desire for more trips or longer trips, with many asking throughout the interviews about when they could next attend a trip. It was evident that a few patients were unaware of some trips. One patient described how he left for his homelands on his own, and after a few days had started to become unwell, but to his relief the bus happened to be visiting, which meant he could receive treatment without aeromedical evacuation.

#### Bus attendance: Quotes

P2: “*It’s really disheartening how the trips have gone from nine weeks [of the year] to only two. We just have to sit here on the machine, getting disheartened.”*

*P14: ‘When is it coming up though? Coz it’s nearly Christmas time now and I wanna go to Christmas to see my family but no renal. I want to sit down with my happy family’.*

*P6: ‘Last time I was at a funeral, I was in Pipalyatjara.. and I saw the truck there. Nobody told me! The community nurse told me. She said “hey that dialysis truck is there!” (laughing), I said “hey, I never heard from there!”, then I did four hours in the truck there I was really happy.’*

Patients identified positive aspects of the trips reflecting the service to be safe and comfortable. All participants reported good relationships with the staff on the trip, P3 relayed that ‘*they’re good people’*, and P11 described the staff ‘*like family*’. The fact that there were three chairs on the bus was also identified by patients as a facilitator, meaning up to 12 patients could attend any one trip.

### Theme 4: Happiness and quality of life

The patients described many benefits of attending dialysis bus trips, particularly by assisting them to fulfil cultural commitments, allowing them to participate in culture and be on Country. Patients described being happy because of the ability to connect with family and see their land, allowing them to participate in the activities that they would otherwise have missed out on due to their health needs, and to visit culturally significant places such as watering holes, mountains and trees. One patient wanted to attend council meetings to have a say on community matters. The staff also consistently identified how the bus provided improved social and emotional wellbeing to patients. This was evident when staff saw their patients meeting their families and engaging in cultural activities. The degree of emotional and social wellbeing improvement of patients, derived from being on the bus, was described by staff as a type of “transformation” by several staff members.

#### Happiness and quality of life: Quotes

P2: *“The bus is really good for us, it gives us a chance to get home so we can have a voice”*.

P6: *‘it is really good to see the whole family and that place. That feeling make me happy’*.

S8: *“It was just amazing seeing the look on his [patient’s] face when he got home … the look on his face when the grandkids ran out and you know, it was just priceless”.*

S4: “*They were transformed. They were completely different to what I see or saw three times a week, up there. Completely different”*.

A major benefit identified by patients was that they could safely dialyse on their homelands and avoid urgent evacuation to hospital. It gave them an alternative to returning to their communities without dialysis support. This was dependent on the flexibility of timing of trips, and occasionally patients would have a funeral to attend without access to the bus. Staff also noted that the bus represented a motivation for patients to engage with renal services so that they may have access to the trips. The dialysis bus provided an attractive alternative to returning to Country on their own, without dialysis access.

#### Happiness and quality of life: Quotes

P3: *‘last week I went to Amata for a funeral. I left on Wednesday, on the bush bus. There was no renal there. … I got sick and I flew in, the flying doctors had to send me back’.*

S2*: “People did rely on the trips, so the bus helped a lot … people really enjoyed having the time off and not getting their treatment and missing dialysis and getting sick and you know, so, it helped in so many ways”.*

Lastly, the bus increased the capacity of patients to be teachers of Indigenous culture for the younger generations as well as to their nurses, nurturing shared understandings and trust. This was of particular importance to community Elders who felt they were unable to pass on valuable cultural information to the younger generations. Patients could also pass on health information to their young ones; in particular, about their dialysis stories. The children in the communities were also given the opportunity to see inside the dialysis bus and form an understanding of what dialysis is.

#### Happiness and Quality of life: Quotes

S3 *“[children were] keen to see what was going on they were very excited about it all and uh, every child at the school had a relative on dialysis at some stage or another so it was all very exciting for them”.*

P1: *‘I say to them, you gotta eat right, you gotta look after yourself, or else you end up like me, stuck to the chair’*. [A patient describing talking to younger generations about his health].

### Theme 5: Enhanced relationships

A key theme emerging from interviews with staff was that of enhanced relationships which had implications for building trust and better communication between staff and patients, thus improving patient engagement with the dialysis service. The relationship between staff and patients was described as being ‘levelled’ following the shared experience on the dialysis bus trips, and it appeared that this was mostly a function of patients being back in their own environment. Staff also described how patients were more likely to engage in conversations around their health. Staff also felt that, through shared understandings cultivated during the trips, a deeper appreciation for the dislocation that the patients have suffered meant that staff were more comfortable and understanding when caring for their Indigenous patients in the dialysis centres.

#### Enhanced relationships: Quotes

S1: *“you get to know the patients and they have a bit more of a trust and share a lot more. So you become a lot more aware of what’s important to them, and the cultural significance of returning home and getting a connection with Country … and family”.*

S3: *“the relationship was better because they felt more at home in their surroundings, and I felt less at home with my surroundings. So I was out of my comfort zone and they were back in theirs”.*

S4: *“They also would listen to you more about their health, because we had gained a different rapport, a different relationship and perhaps a bit more trust.”*

S2: *‘they (the staff) are a lot more happier to look after the Indigenous patients because they think that they understand a little but more every time they do it’.*

Many staff felt privy to meaningful cultural experiences, which also conferred a positive attitude to work back in the satellite unit. The cultural experience was described as far more valuable than other forms of cultural training. As mentioned in theme 4, the trips increased the capacity for patients to act as teachers of culture, giving them a sense of pride, and providing staff with a reference for how different life is in remote Australia. Nurses were interested in learning where their patients had come from, and this new shared understanding provided a basis for increased trust and engagement.

#### Enhanced relationships: Quotes

S1: *“the staff are a bit more invigorated … a bit more enthusiastic … and [felt] pretty lucky that [they] can go and have that experience”.*

S9: *“We had one guy who took us out to the APY lands where you could only go with an Elder … he took us to places where you can’t go unless you go with an Elder, and he took us out there and explained everything”.*

Not only did the experience of working on the bus improve relationships between staff and patients, but also between rural and metropolitan staff. It also helped forge mentorships between junior and senior staff. Rural and metropolitan staff worked together on trips enabling the exchange of experiences. Rural staff members described improved exchange of information between centres after engaging with the program. Staff from within the same unit who were on trips noted a sense of camaraderie following trips together.

#### Enhanced relationships: Quotes

S7: *“part of the joy of going is that you meet people you haven’t worked with before and you can see how, learn how different areas work”.*

*S1: “I think someone always come back from the trip with something that they’ve learnt from the Adelaide staff and I hope that the Adelaide staff go back the same”.*

*S6: “It was good to show other nurses different ways of doing things, different needling techniques”.*

*S4: “Relationships between the staff definitely grew”.*

### Theme 6: Facilitators to success

Several factors contributed to the success of the bus, including supporting staff’s ability to provide both clinical and technical backup, and the presence of a clear co-ordinator role. Beyond these the use of local knowledge, flexibility and cultural awareness were important commodities. Channels of clinical support were available on trips, with medical and technical support via mobile and satellite phone. The dialysis bus always made contact with the local primary care clinic on arrival, this provided another avenue of support if required. All staff recognised the need for a dedicated bus co-ordinator position. This position added continuity for building and maintaining relationships between satellite units and communities, ensuring the bus visited at appropriate times. Other than acting as a community liaison, the bus co-ordinator also arranged transport for staff and patients to and from the communities, ensured the truck was stocked with enough supplies for dialysis and medications, and would trouble-shoot other issues as they arose, for example blown tires and missing patients.

#### Facilitators for success: Quotes

S7: *“if we weren’t sure [about the clinical state of a patient] then we’d always let the clinic know and then we could get the clinic nurses to see them as well, and so if they needed antibiotics or something then they could get them, and we also had close contact with the rural CPC and [a nephrologist]”.*

S2: “*We need a dedicated person to run the bus so they know exactly what they’re doing and they have a constant communication with particular communities”.*

S4: *“When I had four or five going up together, that was actually quite time consuming… bargaining for tickets and money for them um, yeah it was a bit tricky. … once the co-ordinator had developed a relationship with getting the funding and working all of that out, it made it easier.”*

The need for cultural awareness was identified by staff as vital for maintenance of trusted relationships with the communities they visited for ongoing trips. There was no cultural training other than assumed knowledge from working with Indigenous patients at satellite units. Staff identified that this training might have improved relationships between the service and community. The staff recounted near misses with cultural and language misunderstandings. The use of local knowledge was also identified as a tool that helped the staff find patients when dialysis was due in the communities, and identify safe roads to travel in certain weather conditions.

#### Facilitators for success: Quotes

S6: *“I was able to explain that it was just a miscommunication. But it was lucky that I was able to explain that in that situation otherwise things could have escalated and could have damaged our relationship with the community”.*

S1: *“cultural teaching definitely has to improve”.*

S8: *“we do rely on our patients’ local knowledge coz we had a mix up in Coober where we did realise someone had come up … and we had no idea where they were”.*

The need for flexibility was identified for success of the program, both in the ability to be fluid with concepts of time and meeting points during trips, as well as flexibility in the bus’s visit timetable and scheduling of trips. Examples of this flexibility includes changing scheduling to enable patients to attend funerals, or adapting to accommodation issues (e.g. one staff member had to sleep on a couch on one trip). If patients were not able to attend dialysis promptly, the timing of dialysis had to be altered - staff had to relinquish the rigidity of urban practice regimes in this setting.

#### Facilitators for success: Quotes

S5 *“you have to be very open, and um… change. There’s a lot change, in very short times; you have to be flexible”*.

*S1: “You can’t always pre-plan things … the clients always say ‘can you organise the bus we’ve got a funeral on or we’ve got two funerals’ … and it’s just, it’s not logistically possible but it would be good if there was some way to have a little bit more fluid, the way the bus works … you can’t be rigid in your routines”.*

### Theme 7: Barriers to success

Barriers to the success of trips included external forces such as weather conditions and staffing issues. Environmental considerations were identified as affecting trips but were not modifiable, including wet weather along dirt roads and hot weather. Nurses relayed difficulties surrounding expectations placed on them for certain roles, adding strain on some who already felt culturally and geographically isolated. Expectations included: working longer hours with fewer breaks; having to drive around communities to find patients to collect for dialysis; and, performing dialysis in a remote location on patients who may be from other satellite units. Nurses described feeling either intimidated or uncomfortable carrying out some of these roles. Organising time to be off of work during bus trips was also felt to be difficult with pressures from short staffing.

#### Barriers to success: Quotes

S10: *“[The co-ordinator] had to sleep on the lounge floor a couple of times because there wasn’t enough accommodation for all of them to stay”*.

S4: [when a tire exploded] *“a bit scary on the side of the road in the middle of nowhere”.*

*S7: “It can be a little bit intimidating, like I say there’s two females and you’re in the community picking up the patients and you’re knocking on random doors, that can be a little bit intimidating”.*

*S7: “Unfortunately we’ve got a pool of nursing staff who are trained in … dialysis, so you don’t have an excess of that, so for example you can’t take two nurses from one unit for the truck at the same time, they’ve still got sick leave and annual leave and you know, long service leave that they then have to backfill”.*

## Discussion

This study is the first to explore the perceived benefits and accessibility of a mobile health service for Indigenous people. Whilst the inequities of health service access for Indigenous populations are well described in Australia [34], New Zealand [35, 36], Canada [37–39] and the US [40], few studies have looked at health services combatting this inequity [11]. Negative effects of separation from Country were identified. Grief associated with dislocation from kinship, land and culture was compounded by a loss of community presence and ‘a voice’, following being confined to the ‘Wrong Country’. The dialysis bus has been shown to benefit patients by providing a means to fulfil cultural commitments; an alternative to skipping dialysis to return to Country; and a platform to build trust and relationships between patients and staff. The dialysis bus also provided a valuable cultural learning opportunity for staff. Successful trips were also characterised by access to clinical and technical support staff and the bus co-ordinator role.

### Benefits of the bus

The findings of this study illustrate how a mobile health delivery strategy can improve the social and emotional wellbeing of Indigenous ESKD patients dislocated from their Country, which has significant clinical implications. The health and wellbeing of Indigenous Australians are strongly connected to land and family [41] and the consequences of removal from this are well documented [42]. Similar connections to land and health are identified for other Indigenous peoples in Canada [43], with similar disparities between rural and remote dwellers on their incidence of CKD [44], identifying a need for better access to specialist health services in geographically isolated settings. The dialysis bus was consistently reported to have positive impact on patients, providing them an opportunity to return home to participate in Country and culture and spend time with family, alleviating the pain and grief from separation and displacement. The authors were unable to find data on the benefits of a rural mobile health delivery service, or on ways of alleviating the grief associated with the displacement that comes with relocating for dialysis. Whilst there are two dialysis bus services in Australia that provide this service [24], and one mobile dialysis service that provides a similar service in Canada [23] and Australia [22], evaluations have not been reported. This study provides initial insight into the benefits of such a service, as well as examining the ways that it operates and succeeds.

### Bus as a service

International literature describes the importance of the use of Indigenous knowledge, community engagement and consultation in the success of Indigenous health services [11, 19, 45, 46]. Self-determination, including the right to self-govern health services, has been recognised as a core ingredient of improved service delivery and access [19]. This study identified that the use of local knowledge contributed to the success of trips; however, routine use of local knowledge was not identified as part of the service. A recent review examining health care delivery for Indigenous populations described how community engagement was associated with improved health outcomes and has been shown to foster self-management [47].

Patients in our study felt the bus was a welcoming space. This is an important concept which has been shown to be associated with improved treatment and service uptake [48]. Patients were more confident and talkative after time on Country, relating to the physical space on the bus as well as the emotional and relational aspects of the service [48]. The relationship between healthcare providers and Indigenous patients has been shown to be crucial to the decision of the patient to engage with a service [48], and this study shows how the bus fosters increased trust and mutual respect between staff and patients. Staff felt they had improved relationships and more effective communication with patients following shared experiences on the trips, leading to increased engagement with staff in health discourse. The building of patient-provider relationships has been shown to improve service accessibility in the literature [49], and enhanced communication has been shown to improve care accessibility for Indigenous people [11]. Poor communication has been identified as a major problem in Indigenous health care. An Australian qualitative study investigating effectiveness of communication in an Australian renal unit showed significant miscommunication between staff and patients and their carers on issues such as diagnosis, treatment options and prevention [50]. Poor communication has been linked to patients’ poor understanding of their own disease, with consequent reduced self-management and adherence to medical treatment [51]. Effective communication has been shown to correlate with improved health outcomes [52].

Importantly, the bus was shown to provide patients with an opportunity to pass on cultural knowledge to young ones and teach them about health and kidney disease. Elders of communities, in particular, relished the ability to engage in knowledge sharing on trips, demonstrating how the bus has the capacity to provide health promotion as well as health delivery. Having respect for the role of community Elders on Country has also been shown to improve success of Indigenous primary health services worldwide [53–55].

Staff benefits included improved relationships between staff and an important opportunity to learn from one another. Their cultural learning was also precious and felt to be worth far more than any prior cultural exercise. Few existing cultural training modules are known to have been successful in ensuring acceptable culturally safe care [56]. This newfound cultural knowledge was of particular value as it was able to be utilised back in satellite dialysis units, showing how one service has the capacity to improve the cultural safety of other services. Culturally safe care is central and pivotal to the success of Indigenous health services [19, 49].

Access was limited for patients who no longer had accommodation in their homelands. This inequity relates strongly to the social determinants of health as important barriers to health care in this population [57]. Studies show that availability of transport is important for treatment uptake for Indigenous people [58] and this is not provided by the dialysis bus service. Health services that engage in addressing the social determinants of health by providing a more holistic approach to care are more successful comprehensive programs for Indigenous people [59, 60]. Whilst the bus service is not designed to provide primary health care, it may be able to provide a more accessible service by assisting in providing transport and accommodation for those that need it.

Fluid concepts of time supported partnerships between Indigenous and non-Indigenous groups [61] and flexibility with time was identified as contributing to successful trips**.** Technical and clinical backup was successful and aided in the provision of a flexible service. Such approaches to care have been identified as a characteristic of successful Indigenous primary health care models [19]. Short staff planning was identified as a barrier to trips. Some staff were overwhelmed by role expectations. This deterred them from returning to take further trips, and they needed support in order to overcome incidental issues such as missing patients and blown tires [62]. The need for a co-ordinator was strongly identified by staff as a vital ingredient to the success of the bus, as consultation and consistent communication are important for success of programs occurring in a range of different communities, each with their own needs [48, 59, 60, 63].

The dialysis bus takes into account the social and cultural elements of health care delivery, and acknowledges that positive health care encounters are founded on trust of providers, building on the awareness of the broader factors that affect engagement of services by Indigenous patients.

### Limitations and future recommendations

Whilst this study provides new insights from the patients about a service from a qualitative perspective, it is acknowledged that, in some instances, data quality was limited by the language barrier between the researcher and the patient; with limited access to interpreter services. Further data could have been sought in the form of field notes from attending bus trips; however, time constraints meant that this was not possible and data was relied upon from interviews and field notes during interviews. Member checking would also add to the rigour of data; however, this was technically difficult given geographical distances.

Potential areas for future research could include collecting data from patients who had not been on trips, to elicit their experiences and compare data on quality of life. Quantitative data could also be sought on hospital admissions and RFDS retrievals as the data from this study suggests that these are reduced in the presence of the dialysis bus. Given suggestions that engagement is also improved, biomedical data on dialysis encounters could be sought to see if adherence to dialysis and associated management regimes also improve for patients who have spent time on the dialysis bus.

## Conclusion

This qualitative study evaluating the dialysis bus has shown the program to be beneficial for the social and emotional wellbeing of Indigenous patients who have had to relocate for dialysis. The bus is shown to build relationships with nurses and patients and their communities, potentially conferring better engagement with renal services. The dialysis bus also was shown to be beneficial to nurses in terms of cultural knowledge and competency with a privileged experience to learn from their patients.

Recommendations arising from this study are directed at modifying barriers noted by staff and patients towards the success of trips on the dialysis bus. This includes the arrangement of appropriate transport and accommodation for patients that need it:

- Successful communication to patients, particularly those who are far from Adelaide in Alice Springs for advertisement of trips.
- The need for a champion of the bus, a co-ordinator who manages the above and liaises with communities on a continual basis to ensure the communities are prepared for trips.
- The use of Indigenous local knowledge for cultural understandings and practical knowledge of the land and roads for the bus, increasing consultation and capacities for Indigenous people.
- Using the bus as a health promotional tool for CKD prevention for families and patients in remote communities.

## Additional file

Additional file 1: Interview Guide, List of interview questions used to guide interview. (DOCX 12 kb)

## Abbreviations

APY: Anangu Pitjantjatjara Yankunytjatjara
ESKD: End stage kidney disease
NT: Northern Territory
SA: South Australia
