# Supplementary material for: Indigenous patient experiences of returning to country: a qualitative evaluation on the Country Health SA Dialysis bus
Source: BMC Health Serv Res. 2018 Dec 29;18:1010. doi: 10.1186/s12913-018-3849-4 (PMC6311048; doi:10.1186/s12913-018-3849-4)
Supplement: Supplementary file 1 — Interview Guide, List of interview questions used to guide interview. (DOCX 12 kb) [file 12913_2018_3849_MOESM1_ESM.docx]

**Interview Questions Guide**

**For patients:**

When did you first start having kidney trouble? How did it make you sick?

When did you start dialysis? How do you find dialysis?

Tell me about the time that you started dialysis.

Where did you start the dialysis?

Did you have to move away from home?

(if yes) How do you find living away from home?

Have you ever missed out on dialysis?

Have you ever had to fly on the plane to get back to town for dialysis?

What are the good things about dialysis?

What are the bad things about dialysis?

How did you hear about the dialysis bus?

What do you think about the bus?

What is it like being on the bus?

What do you like about the bus? How come?

What don’t you like about the bus? How come?

What’s it like going home?

What sorts of things do you do when you visit home on the dialysis bus?

If you could change something about the bus – what would it be? Why?

What do you think of the staff on the dialysis bus?

How did the community react when you came home on the dialysis bus? How come?

How did your family react when you came home on the dialysis bus? How come?

Have you had any trouble with the dialysis bus? / Has anything gone wrong while you were on the dialysis bus? What happened?

**For nursing staff:**

How did you come to work on the mobile dialysis unit?

In what ways are working on the mobile unit different to the satellite unit?

How does it impact your relationship with the patients? If so, why do you

think that is?

Do you notice any differences in the wellbeing of patients on the bus? If so,

why do you think that is?

What do you see as the good parts of the bus from your perspective? Why?

Have you had any problems on the dialysis bus? If so, what occurred?

Is there anything you would change about the way the bus functions? Can you describe what you would do differently?
